# Are Sitting Occupations Associated with Increased All-Cause, Cancer, and Cardiovascular Disease Mortality Risk? A Pooled Analysis of Seven British Population Cohorts

**DOI:** 10.1371/journal.pone.0073753

**Published:** 2013-09-26

**Authors:** Emmanuel Stamatakis, Josephine Y. Chau, Zeljko Pedisic, Adrian Bauman, Rona Macniven, Ngaire Coombs, Mark Hamer

**Affiliations:** 1 Physical Activity Research Group, Division of Population Health, University College London, London, United Kingdom; 2 Department of Epidemiology and Public Health, University College London, London, United Kindom; 3 Prevention Research Collaboration, School of Public Health, University of Sydney, Sydney, Australia; Hunter College, City University of New York (CUNY), CUNY School of Public Health, United States of America

## Abstract

**Background:**

There is mounting evidence for associations between sedentary behaviours and adverse health outcomes, although the data on occupational sitting and mortality risk remain equivocal. The aim of this study was to determine the association between occupational sitting and cardiovascular, cancer and all-cause mortality in a pooled sample of seven British general population cohorts.

**Methods:**

The sample comprised 5380 women and 5788 men in employment who were drawn from five Health Survey for England and two Scottish Health Survey cohorts. Participants were classified as reporting standing, walking or sitting in their work time and followed up over 12.9 years for mortality. Data were modelled using Cox proportional hazard regression adjusted for age, waist circumference, self-reported general health, frequency of alcohol intake, cigarette smoking, non-occupational physical activity, prevalent cardiovascular disease and cancer at baseline, psychological health, social class, and education.

**Results:**

In total there were 754 all-cause deaths. In women, a standing/walking occupation was associated with lower risk of all-cause (fully adjusted hazard ratio [HR] = 0.68, 95% CI 0.52–0.89) and cancer (HR = 0.60, 95% CI 0.43–0.85) mortality, compared to sitting occupations. There were no associations in men. In analyses with combined occupational type and leisure-time physical activity, the risk of all-cause mortality was lowest in participants with non-sitting occupations and high leisure-time activity.

**Conclusions:**

Sitting occupations are linked to increased risk for all-cause and cancer mortality in women only, but no such associations exist for cardiovascular mortality in men or women.

## Introduction

The health benefits of leisure-time physical activity are well defined through several decades of epidemiological and clinical studies [1]. By contrast, the relationship between occupational physical activity [2] and sedentary behaviours with health outcomes is less clear. The seminal work by Morris and colleagues [3] that compared the risk of CHD in bus drivers with that of active bus conductors was interpreted as a study of physical activity although in essence it was the first study demonstrating the health effects of a sedentary occupation. The term “insufficiently active” denotes not reaching recommendations for moderate or vigorous physical activity, whereas sedentary behaviours are defined as low-energy-expenditure activities (≤1.5 MET) in a sitting or reclining posture, such as computer use, watching television or driving a car [4].The distinction between “insufficiently active” and sedentary behaviours is important, as the health consequences may be different. Although the evidence is not conclusive, several epidemiological studies have shown adverse health effects of various sedentary behaviour indicators, particularly TV viewing, independently of physical activity level [5]–[6].

Studies employing objective measures have reported a very high prevalence of daily sedentary time [7]–[8], with a representative sample of US adults spending 7.3–9.3 hours in sedentary behaviours, which is more than 55% of their waking time [7]. In addition, increasing trends have been determined for sedentary time internationally [9]–[10]. Because of its wide distribution and the significant increment over time, sedentary behaviour is considered a potential global public health problem.

Total sedentary time is comprised of at least four domains: (a) occupational, (b) leisure-time, (c) transport-related, and (d) domestic. Although there is still no strong supporting evidence, the results of previous studies indicate that different domains of sedentary behaviour might show specific relationships with health [11]–[13]. While less research has focused on transport-related sitting, one study showed that time spent riding in the car is a significant predictor of cardiovascular mortality [14]. The occupational and leisure-time domains of sedentary behaviour have received more attention. A recent meta-analysis has shown that television viewing, the most studied type of leisure-time sedentary behaviour, is significantly associated with an increased risk of type 2 diabetes, cardiovascular disease, and all-cause mortality [5], and another systematic review suggested it is associated with depression and low life satisfaction [15]. Studies on occupational sitting and health risks have not provided such definitive evidence. Since the original work by Morris et al [3], that demonstrated increased risk of cardiovascular disease in sitting occupations, a systematic review by van Uffelen et al [16] showed inconsistent or conflicting results for the association between occupational sitting and cardiovascular disease, diabetes, cancer, and body mass index. Besides, the review encompassed four prospective studies on the association of occupational sitting with all-cause mortality [17]–[20], five studies with cardiovascular mortality [17]–[18], [20]–[22] and one with cancer mortality [17]. Findings for both cardiovascular and all-cause mortality were inconsistent across studies. No association of sedentary behaviour with all-cause and cardiovascular mortality was found in one and two of these studies, respectively, while associations with increased risks of both outcomes were found in three studies [16]. The only previous study which linked sedentary behaviour at work and risk of cancer mortality found no association. The results of one prospective cohort study identified since that review found no association between occupational sedentary behaviour and risks of both cardiovascular and all-cause mortality in disease-free adults [23]. Inconsistency in results for cardiovascular and all-cause mortality, and scarce evidence for cancer mortality, precludes definitive conclusions regarding their association with occupational sitting.

Further research on the relationship of occupational sitting and health risks is particularly important because the large majority adults aged 15–64 years are employed [24], many in work environments that require prolonged sitting [25], [26]. Working hours account for over half of total waking time [27]. Studies indicate a decreasing trend for energy expenditure at work [28], and workers in many professions spend on average more than 70% of their work time sitting [29], [30].

Therefore, the aim of this study was to determine the impact of occupational sitting on cardiovascular, cancer and all-cause mortality in a pooled sample of seven British population cohorts. The main hypothesis was that people in sitting jobs have a higher risk for all-cause, cancer and cardiovascular mortality than those in occupations that involve mostly standing or walking. A secondary aim was to examine the combined effect of non-occupational physical activity and occupational sitting on mortality risk.

## Materials and Methods

### Study Sample and Design

Details of the sample design and selection can be found elsewhere [31]. In brief, participants were drawn from the Health Survey for England (HSE) and the Scottish Health Survey (SHS) – a series of seven independent cohort studies with baseline examinations in 1994 (HSE only), 1998, 1999 (HSE only), 2003, and 2004 (HSE only). The two surveys are run by the same research agencies (Joint Health Surveys Unit) and have identical methodologies. The two studies are general population-based, sampling individuals living in households in each country. HSE and SHS samples were selected using multi-stage stratified probability design to give a representative sample of the target populations. Stratification was based on geographical areas and not on individual characteristics: postcode (zip code) sectors were selected at the first stage and household addresses at the second stage.

These analyses used secondary data from the Health Survey for England (HSE) and the Scottish Health Survey (SHS) in multiple survey years. Ethical approval was granted for all aspects of these studies by the following Ethics Committees prior to each survey year data collection: HSE 1994 was approved by the Medical Ethics Committee of the British Medical Association; HSE 1998/99 were approved by North Thames Multi Centre Research Ethics Committee; HSE 2003/2004 were approved by the London Multi-Centre Research Ethics Committee; SHS 1998 was approved by the Research Ethics Committees for All Health Boards for Scotland; SHS 2003 was approved by the Multi Research Ethics Committee for Scotland. Each sampled address for the HSE and SHS was sent an advance letter which introduces the survey and states that an interviewer would be calling to seek permission to interview. A leaflet was also enclosed providing general information about the survey and some of the findings from previous surveys. Individual interviews were conducted with adults who give verbal informed consent. At the end of individual interviews, participants were asked for agreement to a follow-up visit by a trained nurse. There was no formal record that participants have given verbal consent to the individual interview or gave physical measurements that are not biological samples (e.g. height, weight). It was made clear in the advance letters and information leaflets that participation in the survey is entirely voluntary, and that participants may decline to answer individual questions, withdraw or stop at any time, or refuse any particular measurement if they wish to do so. The procedures used in the HSE to obtain informed consent were very closely scrutinised by a National Health Service (NHS) and the Scottish Executive ethics committee each year. Information leaflets and both the content and wording of questionnaires were also reviewed by the ethics committees.

Participants in this study were aged 40 years and over at study induction. In the present analyses we included cohort members with complete data on all required variables who consented to their death being flagged by the National Registry.

### Clinical and Personal Characteristics

Computer-assisted personal interviewing modules assessed respondents’ demographics, self-reported general health and history of disease (cardiovascular disease and cancer; doctor-diagnosed cardiovascular disease), health behaviours (smoking habits; frequency of alcohol intake; physical activity), and socioeconomic characteristics (occupational social class; age completed full-time education as an indicator of socioeconomic status). Psychological health was evaluated using the 12-item version of General Health Questionnaire (GHQ) [32]. In a separate visit, qualified nurses measured waist circumference (at the midpoint between the lower rib and the costal margin) using an insertion tape.

### Physical Activity and Occupational Sitting

Main activity at work (occupational activity) was assessed with the following question: “*When you’re at work are you mainly sitting down, standing up or walking about?*” The (non-occupational) physical activity questionnaire used in these cohorts has been described in detail elsewhere [33] and is summarised here: questions enquired about frequency (number of days in the last 4 weeks), duration (minutes per day) of participation in domestic activity (e.g. housework, “do-it-yourself” (DIY), gardening, restoration work), brisk walking and cycling for any purpose, and any recreational exercise of moderate-to-vigorous intensity (e.g. swimming, aerobics, callisthenics, gym exercises, team sports, racket sports). The criterion validity of the physical activity questionnaire has been demonstrated in a recent study on 106 English adults from the general population (45 men) where the output of accelerometers (worn for two non-consecutive weeks over a month period) was compared to responses to these questions [34].

Non-occupational physical activity was converted into tertiles of MET-hours/week using standard physical activity intensity tables [35] and an established methodology we have repeatedly used in the past [36]–[37].

### Mortality Follow-up

Participants were flagged by the British National Health Service (NHS) Central Registry, who notified us of the date and cause of death where applicable. Diagnoses for primary (underlying) cause of death was based on the *International Classification of Diseases*, Ninth (ICD-9) and Tenth (ICD-10) Revisions. Codes corresponding to cardiovascular disease mortality were 390–459 for ICD-9 and I01–I99 for ICD-10. Codes corresponding to cancer mortality were 1400–2399 for ICD-9, and C000–D489 for ICD-10.

### Data Handling and Statistical Analysis

Due to evidence of violation of the proportional hazards assumption because of the low number of events in the walking-based occupations group, we developed Cox regression models (with months as the time scale) with the standing and walking occupational activity groups combined and mainly sitting work as the reference. Surviving participants were censored at 31^st^ of December 2009 (SHS) or 15^th^ of February 2011 (HSE). We tested for interactions of occupational activity type with sex and non-occupational physical activity by entering an interaction term in the corresponding age-adjusted Cox models for all three outcomes. Due to evidence for sex interactions for all-cause and cancer mortality (both p<0.001) all analyses were stratified by sex. Due to the low number of events in women we repeated a non-sex specific Cox regression with cardiovascular mortality as the outcome and sex entered as a confounder.

All Cox regression models were adjusted for age (model 1), waist circumference, self-reported general health, frequency of alcohol intake, cigarette smoking, psychological health, MET-hours/week of non-occupational physical activity, prevalent cardiovascular disease (angina/stroke/ischaemic heart disease) at baseline, and prevalent cancer at baseline (model 2). To specifically examine the influence of socioeconomic position in the last stage (model 3) we also adjusted for occupational social class (based on the Registrar General’s social occupational classification [38] (I/II, IIINM, IIM, IV/V) and age finished education (15 years of age or less; 16; 17–18; 19 and over). Since linearity cannot be assessed using two data points only, we also calculated the trend p-value using the three-level occupational activity variable with the standing group as a referent. To address our secondary aim we: a) stratified all Cox analyses by non-occupational physical activity level (using the sex-specific median as a cut-off point); and b) developed a variable that combined information on occupational activity (sitting/non-sitting occupations) and non-occupational physical activity level (low/high, using the sex-specific median as a cut-off point) with the four following groups: (1) low non-occupational physical activity & sitting occupation group; (2) low non-occupational physical activity & non-sitting occupation group; (3) high non-occupational physical activity & sitting occupation group; and (4) high non-occupational physical activity & non-sitting occupation group. Using the first of these groupings as a reference we ran Cox regression analyses with all-cause and cancer mortality as outcomes. We did not run such analysis for cardiovascular mortality due to issues relating to the violation of the proportional hazards assumption.

## Results

### Sample Characteristics

Participant characteristics and bivariate associations for the 5380 women and 5788 men aged ≥40 years at baseline who had valid data required for this analysis are shown in Table 1. Both women and men with standing/walking occupations were older, more likely to smoke, be of a lower education level and social class and have poorer health and less likely to be heavy drinkers, compared to those with sitting occupations. Women with standing/walking occupations were more likely to have a higher waist circumference. In both sexes, those with standing/walking occupations were more likely than those with sitting occupations to be physically active. In men only, all-cause mortality rates were higher in the standing/walking occupational activity group than in the sitting group, although there were no such differences in women.

**Table 1:** Descriptive characteristics of women and men aged ≥40 years by main activity type while at work.

WOMEN				
	Sitting* (n = 2155)	Standing/walkingabout* (n = 3225)	*d (95% CI)* †	*p* ‡
Age (yrs)	49.2±6.6	50.0±7.1	0.8 (0.4–1.2)	<0.001
Waist circumference (cm)	82.9±11.6	84.3±11.7	1.4 (0.8–2.0)	<0.001
General health (% fair/bad/v bad)	15.1 (13.6–16.6)	17.8 (16.5–19.1)	2.7 (0.7–4.7)	0.010
GHQ Score (%≥4)	16.1 (14.5–17.6)	14.9 (13.7–16.1)	1.2 (−0.8–3.1)	0.243
Physical activity (% in the top sex-specific half)	50.8 (48.7–52.9)	54.3 (52.5–56.0)	3.5 (0.8–6.2)	0.012
Smoking (% current)	21.3 (19.5–23.0)	27.3 (25.8–28.9)	6.1 (3.8–8.4)	<0.001
Alcohol frequency (%≥5 times/week)	19.7 (18.0–21.4)	15.4 (14.2–16.7)	4.3 (2.2–6.4)	<0.001
Social class (% manual)	12.3 (10.9–13.7)	47.4 (45.7–49.1)	35.1 (32.9–37.3)	<0.001
Education (% finished age ≥17 yrs)	42.5 (40.4–44.5)	29.4 (27.9–31.0)	13.0 (10.4–15.6)	<0.001
Prevalent CVD (angina/stoke/ischaemicheart disease) (%)	1.6 (1.0–2.2)	2.1 (1.5–2.6)	0.5 (−0.3–1.2)	0.243
Prevalent cancer (%)	4.0 (2.8–5.2)	3.8 (2.9–4.8)	0.2 (−1.3–1.7)	0.825
Died from any cause (%)	5.6 (4.6–6.5)	4.8 (4.0–5.5)	0.8 (−0.5–2)	0.208
Died of cancer (%)	3.7 (2.9–4.5)	2.7 (2.1–3.2)	1.0 (0.0–2.0)	0.035
Died of CVD (%)	0.5 (0.2–0.8)	1.0 (0.6–1.3)	0.5 (0.0–0.9)	0.065
**MEN**				
	**Sitting** * **(n = 2458)**	**Standing/walking** **about** * **(n = 3330)**	***d (95% CI)*** †	***p*** ‡
Age (yrs)	50.0±7.4	51.2±7.7	1.2 (0.8–1.6)	<0.001
Waist circumference (cm)	97.4±10.2	96.4±10.3	1.0 (0.5–1.5)	<0.001
General health (% fair/bad/v bad)	13.6 (12.3–15.0)	16.9 (15.7–18.2)	3.3 (1.4–5.2)	<0.001
GHQ Score (%≥4)	9.7 (8.5–10.9)	8.5 (7.6–9.5)	1.2 (−0.4–2.7)	0.130
Physical activity (% in the top sex-specific half)	53.1 (51.2–55.1)	56.1 (54.4–57.8)	3.0 (0.4–5.6)	0.025
Smoking (% current)	18.5 (16.9–20.0)	27.1 (25.5–28.6)	8.6 (6.4–10.7)	<0.001
Alcohol frequency (%≥5 times/week)	30.8 (29.0–32.6)	27.4 (25.8–28.9)	3.4 (1.1–5.8)	0.004
Social class (% manual)	23.7 (22.0–25.4)	63.8 (62.2–65.4)	40.1 (37.7–42.4)	<0.001
Education (% finished age ≥17 yrs)	50.9 (49.0–52.9)	21.8 (20.4–23.2)	29.1 (26.7–31.6)	<0.001
Prevalent CVD (angina/stoke/ischaemicheart disease) (%)	4.4 (3.5–5.2)	4.7 (4.0–5.5)	0.4 (−0.8–1.5)	0.549
Prevalent cancer (%)	2.7 (1.8–3.6)	1.9 (1.2–2.5)	0.8 (−0.3–1.9)	0.143
Died from any cause (%)	7.3 (6.3–8.4)	9.7 (8.7–10.7)	2.4 (0.9–3.8)	0.002
Died of cancer (%)	2.8 (2.1–3.5)	4.7 (4.0–5.4)	1.9 (0.9–2.9)	<0.001
Died of CVD (%)	2.1 (1.6–2.7)	2.6 (2.0–3.1)	0.5 (−0.3–1.3)	0.271

The Health Survey for England and Scottish Health Survey cohorts.

* Mean ± standard deviation for continuous and percentage (95% confidence interval) for categorical variables.

† Absolute value of the difference between sitting and standing/walking about groups and its 95% confidence interval.

‡ p-value calculated using t-test for continuous and two proportions z-test for categorical variables.

### Sitting Occupations and Mortality

Mean±SD follow up was 12.9±3.3 years. In total there were 754 all cause deaths (265 in women), of which 379 (160 in women) were attributed to cancer and 177 (42 in women) to cardiovascular disease. The observed (unadjusted) all-cause, cancer, and cardiovascular mortality are presented in Figures S1, S2 and S3, respectively. In men, observed all-cause and cancer mortality rates were higher in those who reported a standing or walking-based occupation compared to those in sitting occupations. In women, those in sitting occupations had higher cancer mortality rates than those in walking-based occupations. No clear pattern of observed cardiovascular mortality existed for either men or women. The results of the Cox models are presented in Table 2 (women) and Table 3 (men). For women, rates of all-cause and cancer mortality, but not cardiovascular mortality were lower in the standing/walking group compared to the sitting group even after adjustment for all potential covariates. For all-cause and cancer mortality, the hazard ratios were 0.68 (95% CI 0.52–0.89) and 0.60 (95% CI 0.43–0.85), respectively. We repeated analyses after excluding those who reported cancer or cardiovascular disease at baseline but results were virtually unchanged for both men and (Tables S1 and S2). When we repeated women’s Cox models with non-cancer mortality as the outcome (n = 160 events) we found no evidence for an association with work activity type (multivariable-adjusted HR for the standing/walking group: 0.82 (95% CI 0.52–1.27, p = 0.377). For men, rates of cancer mortality were higher in the standing/walking group compared to the sitting group after adjustment for age, self-reported general health, alcohol, smoking, non-occupational physical activity, cardiovascular disease and cancer at baseline with a HR 1.34 (95% CI 1.00–1.80) but additional adjustments for occupational social class and education attenuated this materially (HR = 1.25 [95% CI 0.91–1.72]). Occupational activity was not associated with either all-cause or cardiovascular mortality in men. When we repeated the cardiovascular mortality analysis for men and women combined (Table 4), occupational activity was not associated with the outcome (HR = 1.06 [95% CI 0.75–1.49]).

**Table 2:** Cox regression models for main activity while at work and all-cause/cancer/cardiovascular mortality in women aged ≥40 years who were in employment at baseline (n = 5214).

WOMEN				
	*All-cause Mortality*			
Predominant activity at work	Cases/total n	Model 1† HR (95% CI)	Model 2† HR (95% CI)	Model 3† HR (95% CI)
Sitting	116/2090	1	1	1
Standing/walking about	149/3124	0.76 (0.59–0.97)	0.73 (0.57–0.94)	0.68 (0.52–0.89)
*Trend p* ‡		0.030 (0.087)‡	0.016 (0.051)‡	0.005 (0.017)‡
	***Cancer mortality***			
Sitting	77/2090	Referent		
Standing/walking about	83/3124	0.65 (0.47–0.88)	0.60 (0.44–0.82)	0.60 (0.43–0.85)
*Trend p*		0.007 (0.021)‡	0.002 (0.006)‡	0.004 (0.014)‡
	***CVD mortality***			
Sitting	11/2090	1	1	1
Standing/walking about	31/3124	1.63 (0.82–3.25)	1.74 (0.86–3.51)	1.53 (0.72–3.24)
*Trend p*		0.161 (0.322)‡	0.121 (0.247)‡	0.272 (0.478)‡

† Model 1: adjusted for age; Model 2; also adjusted for waist circumference, self-reported general health, psychological health, frequency of alcohol intake, cigarette smoking, MET-hours/week of non-occupational physical activity, prevalent cardiovascular disease at baseline (angina/stroke/ischaemic heart disease), prevalent cancer at baseline; Model 3: also adjusted for occupational social class (I/II, IIINM, IIIM, IV/V) and age finished educations (15 years of age or less; 16; 17–18; 19 and over).

‡ p-values in brackets correspond to the trend in the cox models when the main activity at work variable is entered in its original form with 3-categories (sitting/standing/walking about).

**Table 3:** Cox regression models for main activity while at work and all-cause/cancer/cardiovascular mortality in men aged ≥40 years who were in employment at baseline (n = 5620).

MEN				
	*All-cause Mortality*			
Predominant activity at work	Cases/total n	Model 1† HR (95% CI)	Model 2 HR† (95% CI)	Model 3 HR† (95% CI)
Sitting	175/2328	1	1	1
Standing/walking about	314/3237	1.13 (0.94–1.40)	1.04 (0.86–1.26)	0.97 (0.78–1.19)
*Trend p*		0.198 (0.410)‡	0.663 (0.908)‡	0.743 (0.944)‡
	***Cancer mortality***			
Sitting	67/2383	Referent		
Standing/walking about	152/3237	1.44 (1.08–1.92)	1.34 (1.00–1.80)	1.25 (0.91–1.72)
*Trend p*		0.013 (0.043)‡	0.047 (0.141)‡	0.186 (0.391)‡
	***CVD mortality***			
Sitting	51/2383	1	1	1
Standing/walking about	84/3237	1.03 (0.73–1.46)	0.99 (0.69–1.41)	0.98 (0.66–1.45)
*Trend p*		0.864 (0.414)‡	0.942 (0.686)‡	0.934 (0.591)‡

† Model 1: adjusted for age; Model 2; also adjusted for waist circumference, self-reported general health, frequency of alcohol intake, psychological health, cigarette smoking, MET-hours/week of non-occupational physical activity, prevalent cardiovascular disease at baseline (angina/stroke/ischaemic heart disease), prevalent cancer at baseline; Model 3: also adjusted for occupational social class (I/II, IIINM, IIIM, IV/V) and age finished educations (15 years of age or less; 16; 17–18; 19 and over).

‡ p-values in brackets correspond to the trend in the cox models when the main activity at work variable is entered in its original form with 3-categories (sitting/standing/walking about).

**Table 4:** Cox regression models for main activity while at work and cardiovascular mortality in men and women aged ≥40 years combined who were in employment at baseline (n = 10,834).

	*CVD Mortality*			
Predominant activity at work	Cases/total n	Model 1† HR (95% CI)	Model 2 HR† (95% CI)	Model 3 HR† (95% CI)
Sitting	62/4473	1	1	1
Standing/walking about	115/6361	1.14 (0.83–1.55)	1.07 (0.78–1.47)	1.06 (0.75–1.49)
*Trend p*		0.415 (0.256)‡	0.660 (0.541)‡	0.745 (0.452)‡

† Model 1: adjusted for age and sex; Model 2; also adjusted for waist circumference, self-reported general health, psychological health, frequency of alcohol intake, cigarette smoking, MET-hours/week of non-occupational physical activity, prevalent cardiovascular disease at baseline (angina/stroke/ischaemic heart disease), prevalent cancer at baseline; Model 3: also adjusted for occupational social class (I/II, IIINM, IIIM, IV/V) and age finished educations (15 years of age or less; 16; 17–18; 19 and over).

‡ p-values in brackets correspond to the trend in the cox models when the main activity at work variable is entered in its original form with 3-categories (sitting/standing/walking about).

We restricted the main analyses among those 5180 women (n = 231 any-cause, n = 136 cancer, n = 40 cardiovascular deaths) and 5552 men (n = 421 any-cause, n = 189 cancer, n = 125 cardiovascular deaths) who were followed up for three years or more. The direction and magnitude of the observed associations were very similar to those in the main analyses (Tables 2 and 3), for example the multivariate-adjusted HR for women in walking/standing occupations was 0.62 (95% CI 0.47–0.83, p = 0.001) for all-cause mortality, 0.56 (95% CI 0.38–0.82, p = 0.003) for cancer mortality, and 1.46 (95% CI 0.68–3.13, p = 0.326) for cardiovascular mortality. As in the main analyses, no such associations were observed in men for any of the outcomes.

We also repeated the above Cox analyses among 2634 women 2160 men who reported ‘never being a regular smoker’. The direction and magnitude of the observed associations were very similar to those shown in Tables 2 and 3, e.g. the multivariable-adjusted HR mortality among women in walking/standing occupations was 0.61 (95% CI 0.40–0.93, p = 0.022) for all-cause mortality and 0.58 (95% CI 0.35–0.98, p = 0.045) and for cancer mortality. As in the analyses of the full sample, no such associations were observed in men for neither all-cause nor cancer mortality. This sub-group analysis could not be performed for cardiovascular mortality due to the low number of such events (n = 12 in women, n = 31 in men) and the subsequent violations of the proportional hazards assumption in the corresponding models.

### Main Activity at Work and Non-occupational Physical Activity

In women, we found evidence for an interaction between occupational activity type and non-occupational physical activity in terms of all-cause mortality (p = 0.011), but not for cancer (p = 0.130) and cardiovascular mortality (p = 0.087). Nevertheless, in the stratified analyses by physical activity level (using sex-specific median as the cut-off point) we found that for both all-cause and cancer mortality, sitting occupations were linked to an increased risk in those women who were in the high non-occupational physical activity group (all-cause mortality for standing/walking occupation HR = 0.46 [95% CI 0.31–0.68], p<0.001; cancer mortality HR = 0.45 [95% CI 0.27–0.74], p = 0.002), but not in the low non-occupational physical activity group (all-cause mortality HR = 0.96 [95% CI 0.65–1.42], p = 0.839; cancer mortality HR = 0.79 [95% CI 0.48–1.30], p = 0.351). These results were virtually unchanged when we restricted the stratified analyses to those women who reported no cancer and no cardiovascular disease at baseline, e.g. the all-cause mortality multivariable-adjusted HR for the high non-occupational activity group was 0.45 (0.30–0.68, p<0.001). There were no differences in the association of main activity at work and mortality by non-occupational physical activity level groups in men.

**Figure 1:**
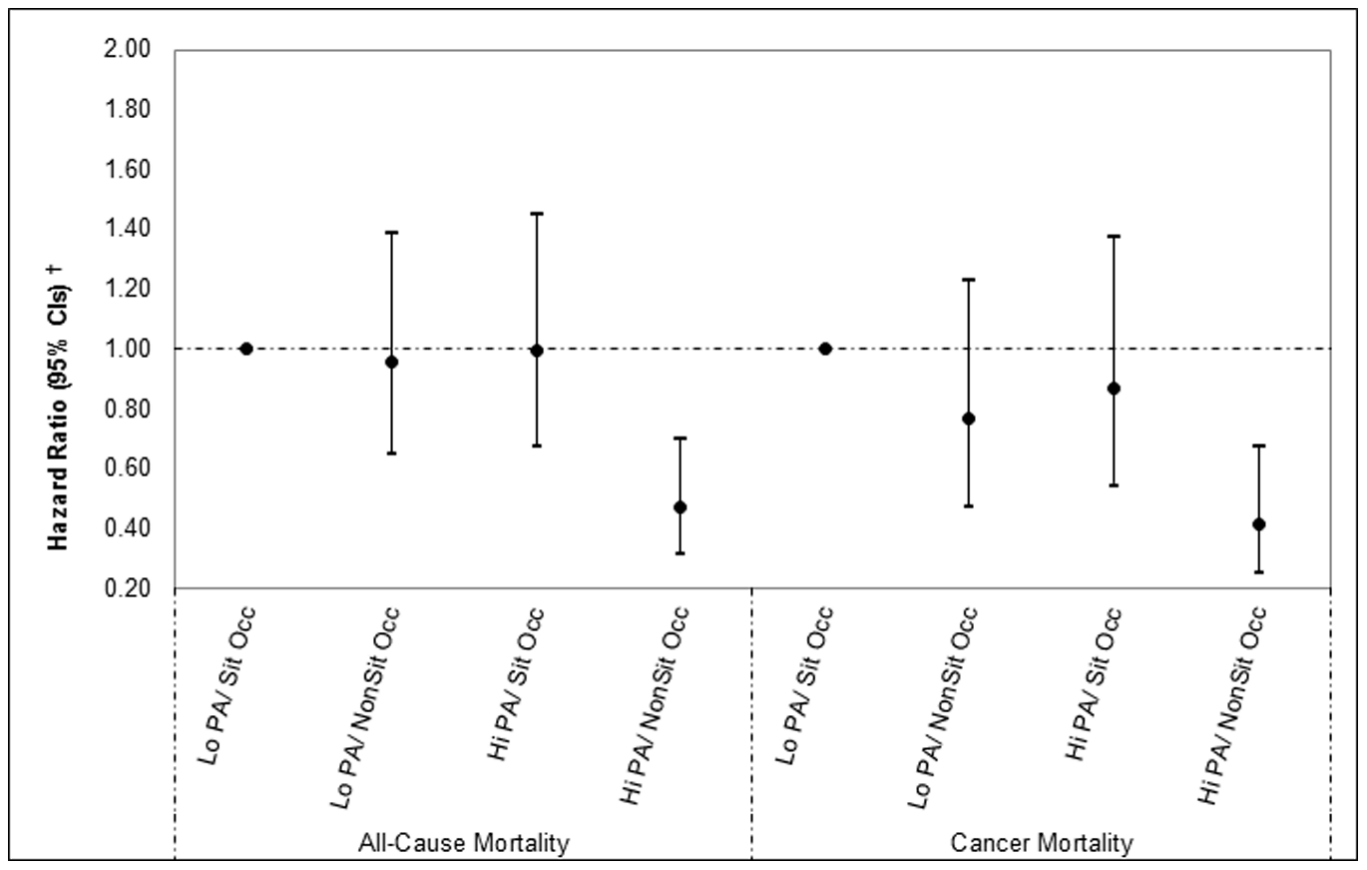
The combined association of main activity at work and non-occupational physical activity in women (N = 5214). Lo PA/Sit Occ: Low physical activity/Sitting occupation; Lo PA/NonSit Occ: Low physical activity/standing or walking occupation; Hi PA/Sit Occ: High physical activity/Sitting occupation; High physical activity/standing or walking occupation. ^†^Adjusted for age, self-reported general health, alcohol drinking frequency, cigarette smoking, prevalent cardiovascular disease at baseline (angina/stroke/ischaemic heart disease), prevalent cancer at baseline, occupational social class and age finished education.

**Figure 2:**
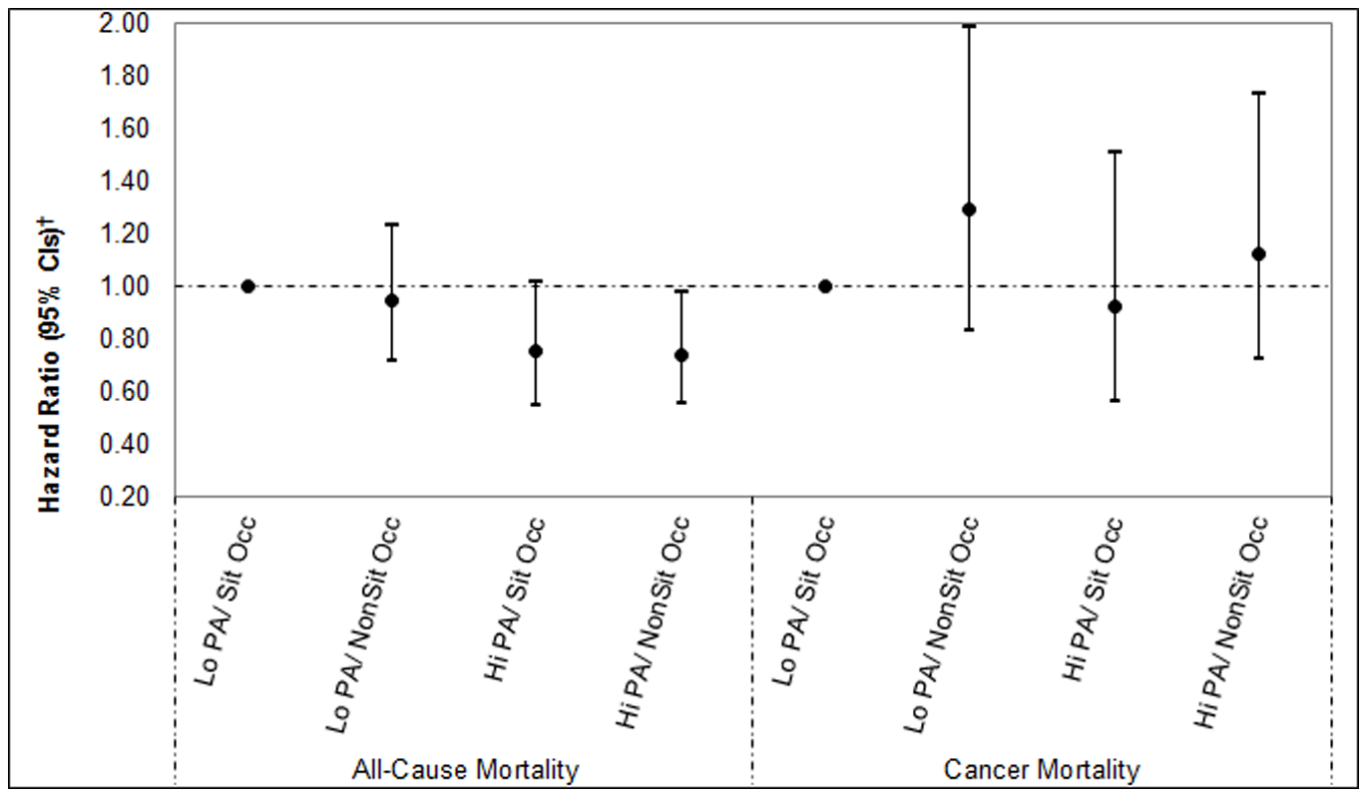
The combined association of main activity at work and non-occupational physical activity in men (N = 5620). Lo PA/Sit Occ: Low physical activity/Sitting occupation; Lo PA/NonSit Occ: Low physical activity/standing or walking occupation; Hi PA/Sit Occ: High physical activity/Sitting occupation; High physical activity/standing or walking occupation. ^†^Adjusted for age, self-reported general health, alcohol drinking frequency, cigarette smoking, prevalent cardiovascular disease at baseline (angina/stroke/ischaemic heart disease), prevalent cancer at baseline, occupational social class and age finished education.

In the analyses with the combined occupational activity and non-occupational physical activity variable as the exposure (Figures 1 and 2), the risk of all-cause mortality was lower in the high non-occupational physical activity/non-sitting occupation group compared to the referent low non-occupational physical activity/sitting occupation group in both women and men. The HRs were 0.47 (95% CI 0.32–0.70) for women and 0.74 (95% CI 0.56–0.97) for men. For women only, the risk of cancer mortality was also lower in the high non-occupational physical activity/non-sitting occupation group compared to the referent group (HR = 0.42 [95% CI 0.23–0.67]), but no association was found in men (HR = 1.12 [95% CI 0.73–1.73]). These results were virtually unchanged when we restricted the stratified analyses to those women and men who reported no cancer and no cardiovascular disease at baseline, e.g. women’s all-cause mortality multivariable-adjusted HR for the high non-occupational physical activity/non-sitting occupation group was 0.44 (0.29–0.67, p<0.001) compared with 0.47 (0.32–0.69, p<0.001) in the original analysis presented in Figure 1; women’s cancer mortality multivariable-adjusted HR for the same group was 0.42 (0.25–0.70, p = 0.001) compared with 0.41 (0.25–0.67, p<0.001) in the original analysis.

## Discussion

The main aim of this study was to examine the association of occupational sitting with cardiovascular, cancer and all-cause mortality risk. We found partial support for our hypothesis that people with jobs involving mainly sitting would have higher risk of all-cause, cancer- and cardiovascular disease mortality compared to people with jobs involving mostly standing or walking.

We found that women with standing/walking occupations had lower risk of dying from all-causes and cancer (by 32% and 40%, respectively), but not from cardiovascular disease, relative to women with sitting occupations, after adjusting for multiple covariates. In men, we found no differences in mortality risk from all-causes, cancer or cardiovascular disease after adjusting for multiple covariates when comparing those in standing/walking occupations with those in sitting occupations.

The findings of the present analyses provide an important contribution to the currently equivocal literature about associations between occupational sitting and mortality risk [16]. Recent evidence suggests that occupations with lower energy expenditure confer higher risk of mortality [2], while others conversely report that high occupational activity is associated with increased mortality risk [39], or that there is no different in risk of death in adults in mostly sedentary jobs relative to those with jobs involving much walking/lifting [23]. Our results indicate that having a mainly sitting occupation is associated with higher risk of mortality from all causes and cancer in women, but not in men. The inconsistencies in this area might be explained by the strong confounding influences of socio-economic status; that is, white collar professional and managerial positions are far more likely to involve sitting at work. Thus the myriad of factors that contribute to better health in individuals of higher social status might offset the effects of their sedentary occupations. In the present study participants in sitting occupations had better health behaviours such as lower rates of smoking, consistent with previous evidence [14]. The fact that sitting might be less strongly associated with social position in women might partly explain the differences between men and women.

The sub-analysis with non-cancer death as outcome suggested that the associations observed between occupational sitting and all-cause mortality in women were driven by cancer-related deaths, while the lack of associations observed between occupational sitting with cardiovascular mortality was likely explained by the relatively small number of cardiovascular disease-related deaths that occurred over the follow-up period. There is fairly consistent evidence that sedentary behaviour [40] and sedentary occupations [41]–[43] are associated with a higher risk of developing some cancers (e.g., colorectal, ovarian, prostate, endometrial); however there is scarce evidence for cancer-related mortality. Nonetheless, our findings are consistent with one systematic review, which concluded that sedentary behaviour is associated with increased risk of cancer-related mortality in women [2].

A secondary aim of this study was to examine the combined effect of non-occupational physical activity and occupational sitting on mortality risk. We found that in men and women with high non-occupational physical activity and non-sitting occupations (high non-occupational physical activity/low occupational sitting), the risk of all-cause mortality was 26% and 53% lower, respectively, compared to those with low non-occupational physical activity and sitting occupations (low non-occupational physical activity/high occupational sitting). Cancer-related mortality risk was significantly lower (by 58%) in women with high non-occupational physical activity and non-sitting occupations relative to those with low non-occupational physical activity and sitting occupations, but no association was observed for men. These results demonstrate the benefits of physical activity in non-occupational domains combined with occupational activity, which have been examined together infrequently. Studies that have examined occupational and non-occupational activity and their associations with mortality separately have yielded similar results [2], [39], However, one study that combined occupational activity with leisure-time physical activity found that higher leisure-time physical activity was associated with lower risk for both cardiovascular and all-cause mortality across different occupational activity levels in both men and women [44]. In contrast, we found that only adults with the high non-occupational activity and non-sitting occupation profile (i.e., the most “healthy” combination of behaviours) had significantly reduced risk of mortality relative to those with the low non-occupational activity and sitting occupation profile (i.e., the least “healthy” combination of behaviours). Further investigation of the combined effects of occupational and non-occupational physical activity on mortality risk would help clarify these observations. Furthermore, future examination of sex differences in associations of non-occupational physical activity with mortality risk may also be warranted. While meta-analytic evidence indicates that associations are stronger in women than in men [2], two more recent studies have been mixed regarding sex differences [39], [44].

The mechanism through which sitting occupations are potentially detrimental to health is unclear. The rodent model-based hypothesis that prolonged sitting causes dramatic reduction of lipoprotein lipase activity (by 80–90%) compared with standing up or ambulating was put forward more than a decade ago [45] but has not been verified in humans. Recent laboratory studies have shown that one day of sitting reduced insulin action in young healthy adults [46]and that short bouts of light-intensity walking to break up continuous sitting are linked with reductions in postprandial glucose and insulin in overweight adults [47], suggesting that sitting may perhaps impair glucose metabolism. Any such effects would be amplified by chronic exposure to work sitting across several years or decades. In addition to these metabolic candidate pathways, the association between sedentary behaviour and cancer may also involve adiposity, inflammation, and sex-hormone related pathways [40].

The main strengths of this study were the large study population pooled from seven independent cohort studies, the prospective design of the analyses with mean follow-up of approximately 13 years, the multiple analytical measures we took to minimise the chances of reverse causality, and the linkage of data with the national death registry with cause-specific details. Our analyses were adjusted for a range of demographic and behavioural variables, although residual confounding from unmeasured factors remains a possibility.

One limitation was that despite the relatively large sample size there was a low number of cardiovascular death events that limits our ability to draw conclusions from the corresponding analyses. The use of self-reported measures of occupational and non-occupational physical activity raises the potential of bias or measurement error. However, the non-occupational physical activity measure has demonstrated sufficient validity against accelerometers [34], while the categorical occupational activity measure is similar to that commonly used in previous cohort studies [16]. Another limitation is that there was no information on non-occupational sitting in these cohorts, but from another recent study (unpublished data) we know that higher socioeconomic position is linked to higher overall sitting but lower TV time. We were unable to examine the potential effects of sedentary behaviour in non-occupational domains as we did for physical activity levels. It is possible that different patterns of associations with mortality may be found if other sedentary behaviour domains were incorporated, such as TV-viewing or daily sitting, both of which have been found to be associated with increased mortality risk [5], [48]. Finally, we had no information on participants’ length of time in their present occupation which potentially introduced some error in our estimates for those who changed type of occupation in the recent past.

The findings of this study have implications against a backdrop of declining trends in daily activity and the increasingly sedentary nature of work. For working populations, occupational time makes up a large part of their day [10], [49] However, daily energy expenditure at work and in other domains of daily living are in decline [9], [28] and have been projected to continue declining over the next decade and a half [9]. We found that women, but not men, with standing/walking jobs had lower risk of dying from all-causes and cancer relative to those with sitting jobs, and also that adults with high non-occupational activity and non-sitting work had lower risk of mortality relative to those with low non-occupational activity and sitting work. Our results support public health initiatives and policies to encourage adults to move more and sit less at work and throughout their day.

## Supporting Information

Figure S1 **Observed all-cause mortality rates by main activity while at work.** Whiskers denote 95% confidence intervals for percentages. *P*-values (group-to-group comparison) were calculated using two proportions z-test.
(TIF)

Figure S2 **Observed cancer mortality rates by main activity while at work.** Whiskers denote 95% confidence intervals for percentages. *P*-values (group-to-group comparison) were calculated using two proportions z-test.
(TIF)

Figure S3 **Observed CVD mortality rates by main activity while at work.** Whiskers denote 95% confidence intervals for percentages. *P*-values (group-to-group comparison) were calculated using two proportions z-test.
(TIF)

Table S1 **Cox regression models for main activity while at work and all-cause/cancer/cardiovascular mortality in women aged ≥40 years who were in employment and reported no cancer or cardiovascular disease (angina/stroke/ischaemic heart disease) at baseline (n = 5027).** ^†^Model 1: adjusted for age; Model 2; also adjusted for waist circumference, self-reported general health, psychological health, frequency of alcohol intake, cigarette smoking, MET-hours/week of non-occupational physical activity; Model 3: also adjusted for occupational social class (I/II, IIINM, IIIM, IV/V) and age finished educations (15 years of age or less; 16; 17–18; 19 and over). ^‡^p-values in brackets correspond to the trend in the Cox models when the main activity at work variable is entered in its original form with 3-categories (sitting/standing/walking about).
(DOCX)

Table S2 **Cox regression models for main activity while at work and all-cause/cancer/cardiovascular mortality in men aged ≥40 years who were in employment and reported no cancer or cardiovascular disease (angina/stroke/ischaemic heart disease) at baseline (n = 5329).** ^†^Model 1: adjusted for age; Model 2; also adjusted for waist circumference, self-reported general health, psychological health, frequency of alcohol intake, cigarette smoking, MET-hours/week of non-occupational physical activity; Model 3: also adjusted for occupational social class (I/II, IIINM, IIIM, IV/V) and age finished educations (15 years of age or less; 16; 17–18; 19 and over). ^‡^p-values in brackets correspond to the trend in the Cox models when the main activity at work variable is entered in its original form with 3-categories (sitting/standing/walking about).
(DOCX)
