# Supplementary figures and images for: Are Sitting Occupations Associated with Increased All-Cause, Cancer, and Cardiovascular Disease Mortality Risk? A Pooled Analysis of Seven British Population Cohorts
Source: PLoS One. 2013 Sep 26;8(9):e73753. doi: 10.1371/journal.pone.0073753 (PMC3784430; doi:10.1371/journal.pone.0073753)

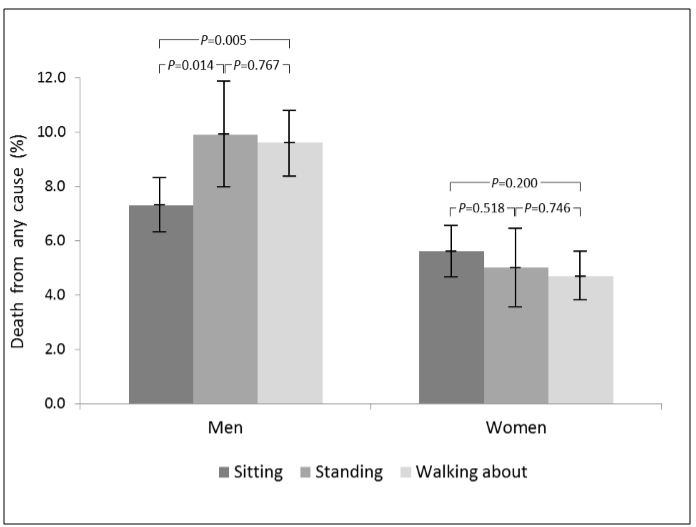

Supplement: Figure S1 — Observed all-cause mortality rates by main activity while at work. Whiskers denote 95% confidence intervals for percentages. P-values (group-to-group comparison) were calculated using two proportions z-test. (TIF) [file pone.0073753.s001.tif]

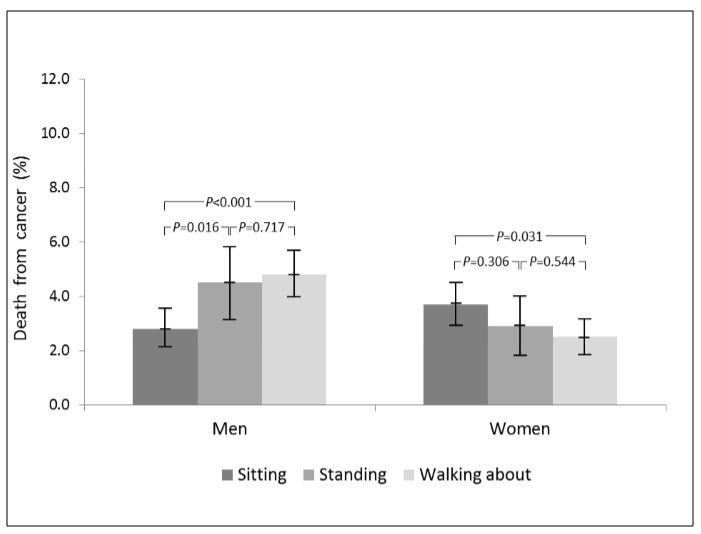

Supplement: Figure S2 — Observed cancer mortality rates by main activity while at work. Whiskers denote 95% confidence intervals for percentages. P-values (group-to-group comparison) were calculated using two proportions z-test. (TIF) [file pone.0073753.s002.tif]

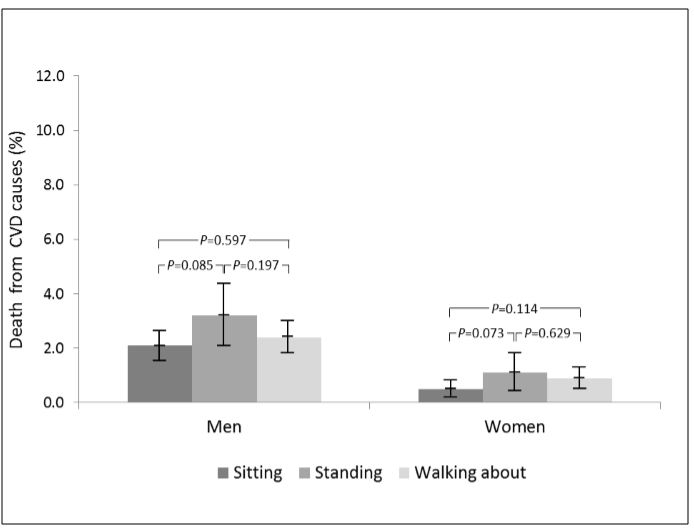

Supplement: Figure S3 — Observed CVD mortality rates by main activity while at work. Whiskers denote 95% confidence intervals for percentages. P-values (group-to-group comparison) were calculated using two proportions z-test. (TIF) [file pone.0073753.s003.tif]
