# Supplementary material for: Are Sitting Occupations Associated with Increased All-Cause, Cancer, and Cardiovascular Disease Mortality Risk? A Pooled Analysis of Seven British Population Cohorts
Source: PLoS One. 2013 Sep 26;8(9):e73753. doi: 10.1371/journal.pone.0073753 (PMC3784430; doi:10.1371/journal.pone.0073753)
Supplement: Table S1 — Cox regression models for main activity while at work and all-cause/cancer/cardiovascular mortality in women aged ≥40 years who were in employment and reported no cancer or cardiovascular disease (angina/stroke/ischaemic heart disease) at baseline (n = 5027). †Model 1: adjusted for age; Model 2; also adjusted for waist circumference, self-reported general health, psychological health, frequency of alcohol intake, cigarette smoking, MET-hours/week of non-occupational physical activity; Model 3: also adjusted for occupational social class (I/II, IIINM, IIIM, IV/V) and age finished educations (15 years of age or less; 16; 17–18; 19 and over). ‡p-values in brackets correspond to the trend in the Cox models when the main activity at work variable is entered in its original form with 3-categories (sitting/standing/walking about). (DOCX) [file pone.0073753.s004.docx]

| **Table S1**: Cox regression models for main activity while at work and all-cause/cancer/cardiovascular mortality in women aged ≥ 40 years who were in employment and reported no cancer or cardiovascular disease (angina/stroke/ischaemic heart disease) at baseline (n=5027) | | | | |
| --- | --- | --- | --- | --- |
|  | | | | |
|  | ***All-cause Mortality*** |  |  |  |
| **Predominant activity at work** | Cases/total n | Model 1^†^ HR (95% CI) | Model 2^†^ HR (95% CI) | Model 3^†^ HR (95% CI) |
| Sitting | 104/2018 | 1 | 1 | 1 |
| Standing/walking about | 137/3009 | 0.77 (0.60 - 0.99) | 0.72 (0.56 - 0.94) | 0.64 (0.48 - 0.86) |
| *Trend p^‡^* |  | 0.045 (0.119)^‡^ | 0.015 (0.050)^‡^ | 0.002 (0.009)^‡^ |
|  | ***Cancer mortality*** |  |  |  |
| Sitting | 67/2018 | 1 | | |
| Standing/walking about | 77/3009 | 0.68 (0.49 - 0.95) | 0.61 (0.44 - 0.86) | 0.59 (0.41 - 0.86) |
| *Trend p* |  | 0.022 (0.063)^‡^ | 0.004 (0.014)^‡^ | 0.006 (0.019)^‡^ |
|  | ***CVD mortality*** |  |  |  |
| Sitting | 11/2018 | 1 | 1 | 1 |
| Standing/walking about | 27/3009 | 1.39 (0.69 - 2.81) | 1.36 (0.69 - 2.78) | 1.07 (0.49 - 2.32) |
| *Trend p* |  | *0.360 (0.540)^‡^* | *0.390 (0.621)^‡^* | *0.860 (0.729)^‡^* |
|  |  |  |  |  |
| ^†^Model 1: adjusted for age; Model 2; also adjusted for waist circumference, self-reported general health, psychological health, frequency of alcohol intake, cigarette smoking, MET-hours/week of non-occupational physical activity; Model 3: also adjusted for occupational social class (I/II, IIINM, IIIM, IV/V) and age finished educations (15 years of age or less; 16; 17-18; 19 and over).  ^‡^p-values in brackets correspond to the trend in the Cox models when the main activity at work variable is entered in its original form with 3-categories (sitting/standing/walking about) | | | | |
