# Supplementary material for: Are Sitting Occupations Associated with Increased All-Cause, Cancer, and Cardiovascular Disease Mortality Risk? A Pooled Analysis of Seven British Population Cohorts
Source: PLoS One. 2013 Sep 26;8(9):e73753. doi: 10.1371/journal.pone.0073753 (PMC3784430; doi:10.1371/journal.pone.0073753)
Supplement: Table S2 — Cox regression models for main activity while at work and all-cause/cancer/cardiovascular mortality in men aged ≥40 years who were in employment and reported no cancer or cardiovascular disease (angina/stroke/ischaemic heart disease) at baseline (n = 5329). †Model 1: adjusted for age; Model 2; also adjusted for waist circumference, self-reported general health, psychological health, frequency of alcohol intake, cigarette smoking, MET-hours/week of non-occupational physical activity; Model 3: also adjusted for occupational social class (I/II, IIINM, IIIM, IV/V) and age finished educations (15 years of age or less; 16; 17–18; 19 and over). ‡p-values in brackets correspond to the trend in the Cox models when the main activity at work variable is entered in its original form with 3-categories (sitting/standing/walking about). (DOCX) [file pone.0073753.s005.docx]

| **Table S2**: Cox regression models for main activity while at work and all-cause/cancer/cardiovascular mortality in men aged ≥ 40 years who were in employment and reported no cancer or cardiovascular disease (angina/stroke/ischaemic heart disease) at baseline (n=5329) | | | | |
| --- | --- | --- | --- | --- |
|  |  |  |  |  |
|  | | | | |
|  | ***All-cause Mortality*** |  |  |  |
| **Predominant activity at work** | Cases/total n | Model 1^†^ HR (95% CI) | Model 2 HR^†^ (95% CI) | Model 3 HR^†^ (95% CI) |
| Sitting | 149/2258 | 1 | 1 | 1 |
| Standing/walking about | 276/3071 | 1.15 (0.95 - 1.41) | 1.04 (0.85 - 1.28) | 0.94 (0.75 - 1.17) |
| *Trend p* |  | 0.157 (0.367)^‡^ | 0.680 (0.853)^‡^ | 0.578 (0.837)^‡^ |
|  | ***Cancer mortality*** |  |  |  |
| Sitting | 60/2258 | Referent | | |
| Standing/walking about | 138/3071 | 1.44 (1.06 - 1.95) | 1.32 (0.96 - 1.79) | 1.19 (0.85 - 1.66 ) |
| *Trend p* |  | 0.019 (0.059)^‡^ | 0.081 (0.205)^‡^ | 0.303 (0.563)^‡^ |
|  | ***CVD mortality*** |  |  |  |
| Sitting | 41/2258 | 1 | 1 | 1 |
| Standing/walking about | 72/3071 | 1.10 (0.75 - 1.61) | 1.06 (0.62 - 1.58) | 1.03 (0.68 - 1.59) |
| *Trend p* |  | *0.640 (0.683)^‡^* | *0.738 (0.937)^‡^* | *0.873(0.949)^‡^* |
|  |  |  |  |  |
| ^†^Model 1: adjusted for age; Model 2; also adjusted for waist circumference, self-reported general health, psychological health, frequency of alcohol intake, cigarette smoking, MET-hours/week of non-occupational physical activity; Model 3: also adjusted for occupational social class (I/II, IIINM, IIIM, IV/V) and age finished educations (15 years of age or less; 16; 17-18; 19 and over).  ^‡^p-values in brackets correspond to the trend in the Cox models when the main activity at work variable is entered in its original form with 3-categories (sitting/standing/walking about) | | | | |
